# Gene-specific selective sweeps in bacteria and archaea caused by negative frequency-dependent selection

**DOI:** 10.1186/s12915-015-0131-7

**Published:** 2015-04-16

**Authors:** Nobuto Takeuchi, Otto X Cordero, Eugene V Koonin, Kunihiko Kaneko

**Affiliations:** Department of Basic Science, Graduate School of Arts and Sciences, University of Tokyo, Tokyo, Japan; Department of Environmental Systems Science, ETH Zurich, Zurich, Switzerland; National Institutes of Health, National Library of Medicine, National Center for Biotechnology Information, Bethesda, USA

## Abstract

**Background:**

Fixation of beneficial genes in bacteria and archaea (collectively, prokaryotes) is often believed to erase pre-existing genomic diversity through the hitchhiking effect, a phenomenon known as genome-wide selective sweep. Recent studies, however, indicate that beneficial genes spread through a prokaryotic population via recombination without causing genome-wide selective sweeps. These gene-specific selective sweeps seem to be at odds with the existing estimates of recombination rates in prokaryotes, which appear far too low to explain such phenomena.

**Results:**

We use mathematical modeling to investigate potential solutions to this apparent paradox. Most microbes in nature evolve in heterogeneous, dynamic communities, in which ecological interactions can substantially impact evolution. Here, we focus on the effect of negative frequency-dependent selection (NFDS) such as caused by viral predation (kill-the-winner dynamics). The NFDS maintains multiple genotypes within a population, so that a gene beneficial to every individual would have to spread via recombination, hence a gene-specific selective sweep. However, gene loci affected by NFDS often are located in variable regions of microbial genomes that contain genes involved in the mobility of selfish genetic elements, such as integrases or transposases. Thus, the NFDS-affected loci are likely to experience elevated rates of recombination compared with the other loci. Consequently, these loci might be effectively unlinked from the rest of the genome, so that NFDS would be unable to prevent genome-wide selective sweeps. To address this problem, we analyzed population genetic models of selective sweeps in prokaryotes under NFDS. The results indicate that NFDS can cause gene-specific selective sweeps despite the effect of locally elevated recombination rates, provided NFDS affects more than one locus and the basal rate of recombination is sufficiently low. Although these conditions might seem to contradict the intuition that gene-specific selective sweeps require high recombination rates, they actually decrease the effective rate of recombination at loci affected by NFDS relative to the per-locus basal level, so that NFDS can cause gene-specific selective sweeps.

**Conclusion:**

Because many free-living prokaryotes are likely to evolve under NFDS caused by ubiquitous viruses, gene-specific selective sweeps driven by NFDS are expected to be a major, general phenomenon in prokaryotic populations.

**Electronic supplementary material:**

The online version of this article (doi:10.1186/s12915-015-0131-7) contains supplementary material, which is available to authorized users.

## Background

Accumulating evidence from ecological and genomic surveys of microbial diversity indicates that archaea and bacteria (collectively, prokaryotes) in nature are organized into genotypic clusters that largely coincide with distinct ecological characteristics [1]. How such patterns of microbial diversity are formed and maintained is an open question in microbial ecology. It is generally agreed that restriction of recombination and balancing selection between genotypic clusters are necessary for stable, sympatric coexistence of multiple distinct clusters [1-4]. More controversial are the roles played by selection and recombination in the formation of such clusters [5,6].

**Figure 1:**
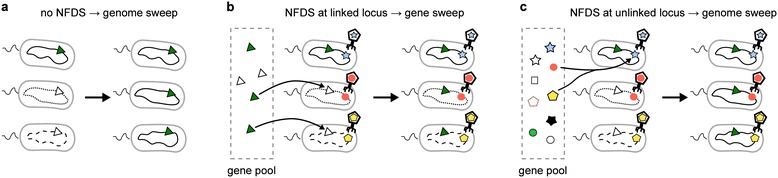
**Schematic diagram showing the mode of selective sweeps under various scenarios.** A closed curve within a cell denotes a prokaryote genome. The different line styles of the genomes indicate the presence of neutral diversity. Green triangles denote the ecologically advantageous allele that spreads through the population. White triangles denote the wild-type allele at the same locus. Other symbols on the genomes denote genes that determine susceptibility to viruses. **(a)** A genome-wide selective sweep occurs in the absence of NFDS without (frequent) recombination. Neutral diversity is lost after the fixation of the beneficial allele. **(b)** A gene-specific selective sweep occurs in the presence of NFDS with recombination only at the locus where the ecologically beneficial allele appears. Neutral diversity is maintained after the fixation of the beneficial allele. **(c)** A genome-wide selective sweep occurs in the presence of NFDS with recombination only at the locus that determines susceptibility to viruses. Neutral diversity is lost. NFDS, negative frequency-dependent selection.

A prominent concept, known as the ecotype model, posits a central role for positive selection and restricted recombination for cluster formation [2]. According to this model, when positive selection causes fixation of a beneficial gene (allele) at one locus in the genome within a population, it also entails fixation at all other loci because recombination is not frequent enough to unlink the beneficial gene from the rest of the genome (Figure 1a). This phenomenon is known as genome-wide selective sweep [7] or genetic hitchhiking [8]. Genome-wide selective sweeps can repeatedly occur in a population adapting to a new environment, each time purging within-population genetic diversity, a phenomenon known as periodic selection [9]. Periodic selection makes a population genetically cohesive and distinct from other populations, leading to cluster formation [5].

Recently, this common view of prokaryote evolution has been challenged by the study of Shapiro et al. [10], which explored the spread of adaptive genes through natural populations of ocean microbes, *Vibrio cyclitrophicus*. This study has shown that adaptive genes spread through microbial populations via horizontal gene transfer (i.e., recombination) without purging genome-wide diversity that was present before the selective sweep. Accordingly, this mode of evolution is denoted gene-specific selective sweep. Under this evolutionary regime, ecologically differentiated individuals are not genetically differentiated at the vast majority of polymorphic loci. Thus, cluster formation rests on establishment of recombination barriers between ecologically differentiated populations [10,11], a situation similar to that of sexually reproducing eukaryotes (e.g., [12-14]). Gene-specific selective sweeps have additional implications for the ecology and evolution of prokaryotes. In particular, under this scenario, genetic diversity within a population is protected from genome-wide selective sweeps that could potentially exert major effects on ecological characteristics of populations such as primary productivity [15]. Moreover, under the gene-specific sweep model, the evolutionary history of a population cannot be described by a single line of succession of common ancestors, in contrast to the implications of the ecotype model [16].

The gene-specific selective sweeps in prokaryotes not only challenge the common view of prokaryote evolution, but also are puzzling with respect to the underlying mechanism. At face value, a gene-specific selective sweep implies that recombination is so frequent as to unlink ecologically beneficial alleles from the rest of the genome before they rise to high frequencies [17,18]. This apparently would require recombination rates far higher than those currently inferred from the available data from many prokaryotes [17,19-22] (see also ‘Discussion’). Although one cannot completely exclude the possibility that current methods underestimate recombination rates by several orders of magnitude, it seems worthwhile to seek an alternative explanation as suggested by the following consideration. The concept of periodic selection, on which the ecotype model is based, was originally derived from experimental evolution of pure bacterial cultures in an isolated environment [9]. Most microbes in the wild do not evolve under such controlled conditions. Rather, they struggle for existence amidst a highly heterogeneous, dynamic ecological community including other evolving microbes, hosts, predators, viruses and plasmids. Ecological interactions with these diverse biological entities substantially impact the course of microbial evolution as indicated by recent work on experimental coevolution [23].

A general mechanism by which such an impact can be made is negative frequency-dependent selection (NFDS), which is a type of selection that favors rare phenotypes in a population [24]. NFDS can be caused by various ecological interactions such as evasion of parasites (also known as the kill-the-winner dynamics) and attack on competitors (e.g., antibiotics production) as well as social interactions such as provision of public goods (e.g., siderophores and virulence factors) [24-28]. NFDS can generate and maintain genetic diversity within a population [29], with the implication that genes adaptive for every individual would spread through a population via recombination, hence gene-specific selective sweeps (Figure 1b) (NFDS has been suggested as a potential cause of gene sweeps by Shapiro et al. [10]; related ideas have been explored by Maynard Smith [30], and Majewski and Cohan [31]; see Discussion). According to this scenario, gene-specific selective sweeps would be a general phenomenon because many, if not most, free-living prokaryotes are likely to evolve under NFDS caused, in particular, by the ubiquitous viruses [32].

A potential problem with the NFDS scenario, however, is that the loci involved in these interactions often are located in genomic islands that appear to experience significantly elevated recombination rates [24,27,33-35]. For example, the O-antigen, the outermost part of lipopolysaccharide protruding from the surface of Gram-negative bacteria, is a typical virus receptor [27]. Genes encoding the O-antigen are likely to evolve under NFDS as attested by their high variability among closely related bacteria [27]. These genes typically are clustered in genomic islands that undergo frequent horizontal gene transfer [36]. In the *Vibrio splendidus* genome, O-antigen-encoding regions contain conserved signal sequences known as JUMP sites, which are exclusively found in these regions and are thought to be involved in natural transformation [37]. Other examples include genomic islands of *Prochlorococcus* cyanobacteria, which encompass genes for various metabolite transporters (potential targets of virus recognition) as well as many tRNA genes, repeat elements and integrases, which can enhance the rate of recombination in these islands [38]. Furthermore, genes encoding various antiviral defense mechanisms, such as restriction-modification systems, also form clusters known as defense islands, which are significantly co-localized with genes encoding transposons and prophage components [35]. Finally, genes encoding synthetases of secondary metabolites that can act as public goods have been shown to appear in mobile regions of prokaryotic genomes. For example, in marine vibrios, toxin-coding genes have been found in genomic islands [39]. Also, secreted virulence factor genes are typically located in the hyper-recombinant regions of many prokaryotic genomes [40]. As a consequence of elevated recombination rates, the loci affected by NFDS can be unlinked from the rest of the genome, and accordingly, NFDS would be unable to prevent genome-wide selective sweeps driven by other adaptive alleles (Figure 1c) [24]. Thus, evaluation of the potential effect of NFDS on fixation of beneficial alleles requires consideration of such biased recombination rates.

Here, we investigate whether and under what conditions NFDS can cause gene-specific selective sweeps in the presence of elevated recombination rates at the loci affected by NFDS. Using mathematical modeling, we show that NFDS indeed can cause gene-specific selective sweeps in large prokaryotic populations, but only when the basal recombination rate is sufficiently low, apparently contradicting the intuition that high recombination rates are required for gene-specific selective sweeps.

## Results

### General framework of the model

**Figure 2:**
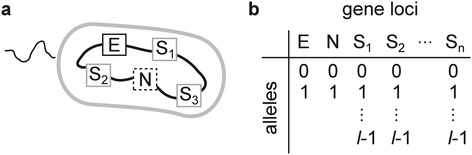
**Conceptual diagram of the model. (a)** A prokaryotic genome assumed in the model. The box labeled with E indicates a locus involved in adaptation to a new ecological niche (E locus). The boxes labeled with S indicate susceptibility loci subject to NFDS (S loci). The box labeled with N indicates a neutral locus (N locus). **(b)** The loci and alleles assumed in the model prokaryote genome. E locus, ecological locus; N locus, neutral locus; S loci, susceptibility loci.

We first introduce the general framework of the model by formulating the question we seek to address (see ‘Materials and methods’ for the details of the model). Suppose that a population of prokaryotes is evolving toward adaptation to a new ecological niche. The genomes of these prokaryotes are assumed to consist of three types of loci (Figure 2a): (i) loci involved in the adaptation to the new niche (E or ecological loci, for short), (ii) *n* loci subject to NFDS (S or susceptibility loci, for short), and (iii) all the other loci, which are assumed to be neutral (N or neutral loci, for short; only one such locus is shown in Figure 2a). The S loci assume *l* alleles per locus, allowing for a total of *l*^*n*^ allelic patterns or *l*^*n*^ susceptibility types (see Table 1 for notation). Each susceptibility type is selectively maintained in the population by the kill-the-winner dynamics. The N loci assume multiple alleles that are selectively neutral. Suppose further that a beneficial allele appears at one of the E loci in one individual of the population. Driven by directional selection, this allele would tend to spread through the population. The question is whether this spread purges genetic diversity at the N loci. If the NFDS is sufficiently strong (see ‘Discussion’ for the justification of this assumption), the frequency of the beneficial allele at the E locus cannot increase beyond the limit imposed by the NFDS, unless the E locus is completely unlinked from all the S loci. Two extreme cases are conceivable that result in such unlinking. In the first extreme case, only the E locus undergoes recombination, whereas the S and N loci are completely linked (Figure 1b). In this case, the beneficial allele at the E locus spreads via recombination without purging neutral diversity at the N loci. Therefore, a gene-specific selective sweep ensues. In the other extreme case, only the S loci undergo recombination, whereas the E and N loci are completely linked (Figure 1c). In this case, the beneficial allele at the E locus can still spread via recombination at the S loci, but the diversity at the N loci is purged because the E and N loci are linked and, thus, sweep together. Therefore, a genome-wide selective sweep ensues (except at the S loci). The above argument illustrates the crux of the problem: whether a gene-specific or genome-wide selective sweep occurs depends on the relative degree of linkage between the E and N loci, and the S and N loci. Thus, we pose the question: If the S loci undergo recombination much more frequently than the E locus (as is likely to be the case), can gene-specific selective sweeps nevertheless occur?

**Table 1:** **Notation**

*α*	Factor by which the recombination rate at S loci is increased from the basal rate *r*
${d}_{S_P,V}$	Number of mismatching loci between *S*_*P*_ and *V*
*E*_*P*_	Allele at the E locus of a host genotype *P*; 0 for WT, 1 for beneficial allele
*f*_*P*_	Fitness of prokaryotic genotype *P*
*f*_*V*_	Fitness of virus genotype *V*
*J*	Clonality at N locus defined as *p*_*N*_^2^ + (1 − *p*_*N*_)^2^
*J*_*a*_	Clonality at N locus after selective sweep
*J*_*b*_	Clonality at N locus before selective sweep
*J*_rel_	Relative clonality at N locus after fixation defined as (*J*_*a*_ − *J*_*b*_)/(1 − *J*_*b*_)
*l*	Number of possible alleles per S locus
*n*	Number of S loci in prokaryote genome
*N*_*P*_	Allele at the N locus of a host genotype *P* (0 or 1)
*P*	Integer representing a prokaryote genotype (0 ≤ *P* < 4*l* ^*n*^)
*p*_*E*_	Frequency of allele 1 at E locus in host population
*p*_*N*_	Frequency of allele 1 at N locus in host population
*p*_*P*_	Frequency of host genotype *P*
*p*_*V*_	Frequency of virus genotype *V*
*r*	Basal recombination rate per locus per generation at E and N loci in hosts
*r*_*V*_	Rate at which allele changes per locus per generation in viral genomes
*s*_*e*_	Selection coefficient of ecologically beneficial allele
*s*_*i*_	Selection coefficient of NFDS imposed at S loci
*S*_*P*_	Allelic pattern of S loci of a host genotype *P* (0 ≤ *S*_*P*_ < *l* ^*n*^)
*V*	Integer representing a virus genotype (0 ≤ *V* < *l* ^*n*^)

E locus, ecological locus; N locus, neutral locus; S loci, susceptibility loci; WT, wild type.

To address the above question, a deterministic population genetics model of evolving prokaryotes under NFDS was developed. The model was formulated as a system of difference equations, ignoring stochastic effects for simplicity (a model incorporating stochastic effects is described in Additional file 1 under the section ‘Effect of finite populations’). The model incorporated NFDS by assuming the kill-the-winner dynamics, following the matching-allele model of infection genetics [41] (an alternative model that incorporates NFDS without explicitly assuming host–parasite interactions is described in Additional file 1 under ‘Alternative model’). For more details, see ‘Materials and methods’.

### Negative frequency-dependent selection can cause gene sweeps when recombination is rare

**Figure 3:**
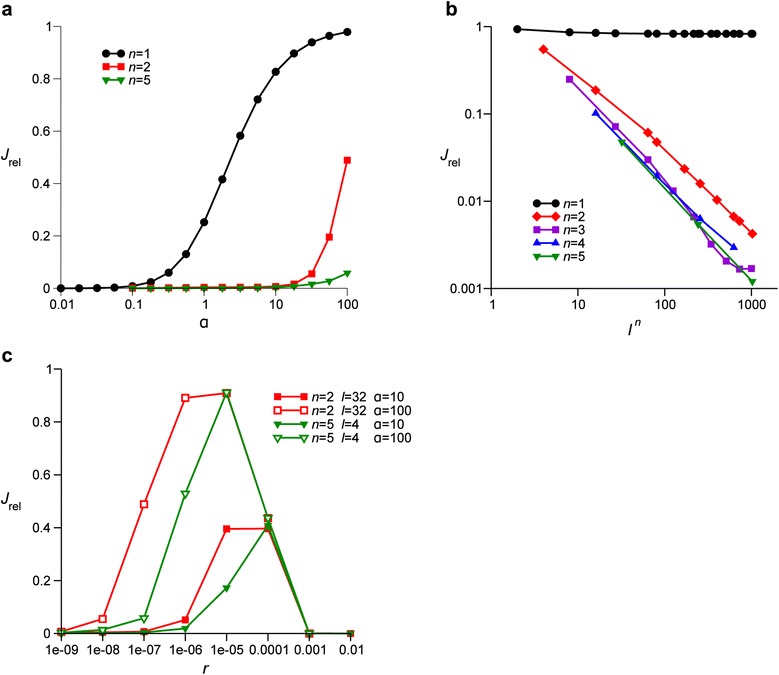
**Relative clonality** ***J***_**rel**_ **as a function of various parameters. (a)** *J*_rel_ as a function of *α*, the factor by which the recombination rate at S loci is increased (or decreased when *α* < 1) over the basal recombination rate at the E and N loci. The value of *l* ^*n*^ was fixed at 1,024, while the value of *n* was varied as indicated in the graph. The other parameters were as follows (see Table 1 for notation): *r* = 10^− 7^, *s*_*e*_ = 0.01, *s*_*i*_ = 0.1 and *r*_*v*_ = 10^− 4^. **(b)** *J*_rel_ as a function of *l* ^*n*^, the number of susceptibility types maintained by NFDS, for various values of *n* as indicated in the graph. The other parameters were as follows: *r* = 10^− 8^, *α* = 10, *s*_*e*_ = 0.01, *s*_*i*_ = 0.1 and *r*_*v*_ = 10^− 4^. **(c)** *J*_rel_ as a function of *r*, the recombination rate at the E and N loci. The value of *l* ^*n*^ was fixed at 1,024, while the values of *n*, *l* and *α* were varied as indicated in the graph. The other parameters were as follows: *s*_*e*_ = 0.01, *s*_*i*_ = 0.1 and *r*_*v*_ = 10^− 4^.

Using the model outlined above, we examined whether gene-specific selective sweeps can occur when recombination at the S loci is substantially more frequent than it is at the E locus. To this end, we measured the relative clonality *J*_rel_ of the population caused by the fixation of a beneficial allele at the E locus for the various parameter values (*J*_rel_ is defined by Eq. (1) in ‘Materials and methods’; see Table 1 for notation). Figure 3a shows *J*_rel_ as a function of *α*, the factor by which the recombination rate at an S locus is increased or decreased over the rate of recombination at the E and N loci (see ‘Modeling recombination’ for the biological rationale of *α*). If the host genome contains only one S locus (*n* = 1), *J*_rel_ becomes almost unity as *α* exceeds one (Figure 3a), indicating that NFDS cannot cause a gene-specific selective sweep when recombination at the S locus is more frequent than it is at the E and N loci. If, however, the host genome contains more than one S locus (*n* > 1), the point of inflection of *J*_rel_ shifts toward much higher values of *α* (Figure 3a). These results indicate that NFDS can cause a gene-specific selective sweep even if the recombination at the S locus is substantially more frequent than it is at the E and N loci, provided the host genome contains at least two S loci.

Figure 3b shows the relative clonality *J*_rel_ as a function of the number of susceptibility types that are selectively maintained at the S loci (i.e., *l*^*n*^) for various values of *n* with *α* = 10. For *n* = 1, *J*_rel_ remains nearly unity as a function of *l*^*n*^, indicating that NFDS cannot cause a gene-specific selective sweep even if it maintains a higher diversity at the S loci as is consistent with the result shown in Figure 3a. For *n >* 1, however, *J*_rel_ is inversely proportional to *l*^*n*^, indicating that the greater the diversity at the S loci is, the more effective NFDS is in causing gene-specific selective sweeps.

Figure 3c shows the relative clonality *J*_rel_ as a function of the basal recombination rate *r* (i.e., the recombination rate at the E and N loci). *J*_rel_ is non-monotonic with respect to *r* and decreases as *r* deviates from intermediate values. This result indicates that a gene-specific selective sweep occurs when recombination is sufficiently rare, apparently contradicting the intuition that high recombination rates are required for gene-specific selective sweeps. A gene-specific selective sweep occurs also when *r* is so high as to be comparable to the selection coefficient of the ecologically beneficial allele *s*_*e*_ (which was set to 0.01). However, in this parameter range, recombination is so frequent that gene-specific selective sweeps occur independently of NFDS [17]. Thus, this parameter range is irrelevant for the key question addressed in this work.

### Interpretation and mathematical analysis of simulation results

To interpret the above results, let us imagine that the prokaryote population is divided into *l*^*n*^ subpopulations. Each subpopulation has an identical allelic pattern at the S loci (i.e., one particular susceptibility type). The frequency of each subpopulation is on average maintained at *l*^−*n*^ by NFDS because all susceptibility types are assumed to be basically the same. Suppose a recombination event brings a beneficial allele into one genome in a subpopulation. This allele spreads through the given subpopulation via clonal expansion of the recipient genome. This expansion, however, is restricted within the subpopulation (the idea is similar to the models described in Maynard Smith [30], Peck [42], Majewski and Cohan [31], and Hodgson and Otto [41]). The smaller the frequency of one subpopulation, the stronger the restriction of clonal expansion. This argument explains why *J*_rel_ decreases in proportion to *l*^−*n*^, which is the average frequency of one subpopulation. Why this result holds only for *n* > 1, is addressed next.

For a beneficial allele to spread beyond any given subpopulation, recombination is required. The relevant recombination events can occur either at the E locus or at the S loci whereby:

- Recombination occurs at the E locus and transfers the beneficial allele into a genome that has a different susceptibility type.

or

- Recombination occurs at the S loci and changes the susceptibility type of a genome that already carries the beneficial allele.

These scenarios differ in their effect on the diversity at the N locus. The scenario involving recombination at the S loci decreases this diversity because it allows clonal expansion of the genome that carries a beneficial allele. By contrast, the scenario involving recombination at the E locus does not decrease the diversity to a similar extent. If *n* = 1, the scenario that involves recombination at the S locus (and thus decreases the diversity) is dramatically more prevalent than the scenario with recombination at the E locus because recombination is assumed to be much more frequent at the S locus than at the E locus (i.e., *α* > > 1 is assumed). In this case, the fixation of the beneficial allele purges diversity at the N locus, resulting in a genome-wide selective sweep (Figure 1c). However, if *n* = 2, the scenario involving recombination at the S loci requires at least two recombination events for the beneficial allele at the E locus to spread throughout the population. To consider this case, let us suppose that a novel genotype with the beneficial allele at the E locus is produced by two successive recombination events at the S loci. The production of this genotype is proportional to (*αrτ*)^2^ where *τ* is the time since the sweep started. The same genotype also can be produced by one recombination event at the E locus. In this case, the production of the genotype is proportional to *rτ*. The timescale of the spread of the beneficial allele is *τ* ~ *s*_*e*_^*-1*^, where *s*_*e*_ is the selection coefficient of the ecologically beneficial allele. Therefore, if ${\left(\alpha r{s}_e^{-1}\right)}^2<<r{s}_e^{-1}$, that is, ${\left(\alpha r\right)}^2{s}_e^{-1}<<r$, the scenario involving recombination at the E locus becomes dominant over the scenario involving recombination at the S loci, leading to gene-specific selective sweeps. This argument explains why *r* has to be sufficiently small for NFDS to cause gene-specific selective sweeps.

The above heuristic argument can be made more precise using a simple mathematical model as described in Additional file 1 (under ‘Maximum recombination rate below which NFDS can cause gene-specific selective sweeps’). Therein, the condition for gene-specific selective sweeps is derived for *n* = 2 as (*ar*)^2^*τ*_2_ < < *r*, where *τ*_2_ is the time required for a beneficial allele to spread through the population. Because *τ*_2_ depends logarithmically on *r* (see Additional file 1), it can be regarded as a constant for an order-of-magnitude comparison between (*αr*)^2^*τ*_2_ and *r*. Numerical calculations indicate that ${\tau}_2>10{s}_e^{-1}$ (data not shown).

**Figure 4:**
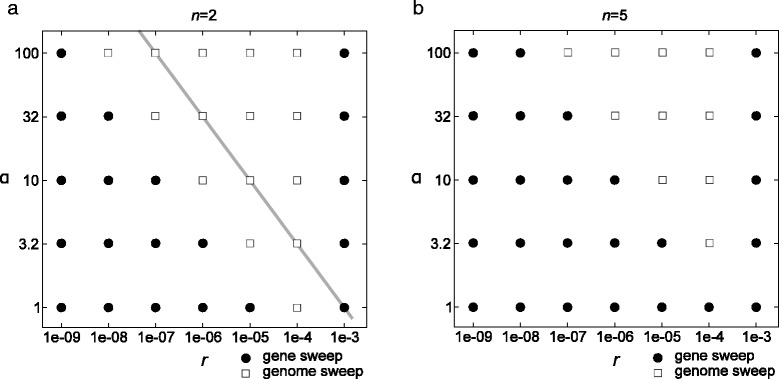
**Phase diagram of the parameter regions in which NFDS causes gene-specific selective sweeps.** Circles indicate a gene-specific selective sweep (*J*_rel_ < 0.05), whereas squares indicate genome-wide selective sweeps (*J*_rel_ ≥ 0.05) for the respective values of *α* and *r*. The parameters were as follows: *l* ^*n*^ = 1,024, *s*_*e*_ = 0.01, *s*_*i*_ = 0.1 and *r*_*v*_ = 10^− 4^. **(a)** *n* = 2. The gray line shows (*αr*)^2^ *τ* = *r*, where *τ* was set to 10*s*_*e*_^−1^ (because ${\uptau}_2>10{\mathrm{s}}_{\mathrm{e}}^{-1}$). **(b)** *n* = 5.

To test the validity of the condition (*αr*)^2^*τ*_2_ < < *r*, we produced a phase diagram displaying the parameter regions in which gene-specific selective sweeps occur (Figure 4a). The results show that the boundary between the parameter regions where gene-specific selective sweeps are caused by NFDS and where genome-wide selective sweeps occur has the same slope as that of the line of (*αr*)^2^ ∝ *r* where the constant of proportionality is arbitrary. This result indicates that the condition (*αr*)^2^*τ*_2_ < < *r* gives a correct power-law relationship between *r* and *α* for the boundary between the two evolutionary regimes, lending validity to the arguments described in the preceding paragraphs. For *n* > 2, the boundary between the two evolutionary regimes is translationary shifted toward a lower value of *r* (Figure 4b), suggesting that the same power-law relationship apparently holds for *n* > 2.

In summary, the condition *n* > 1 ensures that at least two recombination events are required to transform one susceptibility type into the majority of the other susceptibility types. The condition (*αr*)^2^*τ*_2_ < < *r* ensures that the basal recombination rate (*r*) is small enough for processes involving multiple recombination events to be negligible compared with processes involving a single recombination event. Both conditions reduce the effective recombination rate at S loci relative to the basal recombination rate, thus enabling NFDS to cause gene-specific selective sweeps despite the effect of elevated recombination rates at S loci.

### Alternative model without the explicit assumption of host–parasite interactions

Finally, we examined whether NFDS can cause gene-specific selective sweeps regardless of specific mechanisms that cause NFDS. To this end, we considered an alternative model that does not explicitly assume the kill-the-winner dynamics (i.e., host–parasite interactions). In this model, each S locus was subject to NFDS independently. The results obtained with this model were basically the same as described above (see Additional file 1 under ‘Alternative model’), suggesting that gene-specific selective sweeps are independent of specific mechanisms that cause NFDS.

## Discussion

Whether or not NFDS would cause gene-specific selective sweeps is not obvious because of the possibility that elevated recombination rates at loci affected by NFDS could lead to genome-wide selective sweeps. However, investigation of the models presented here indicates that NFDS can cause gene-specific selective sweeps despite this effect, provided two conditions are satisfied. Specifically, there should be at least two loci subject to NFDS, and the basal recombination rate should be sufficiently low. The latter condition is notable as it apparently contradicts the intuition that gene-specific selective sweeps are made possible by high recombination rates.

In addition, there are three conditions that are required for NFDS to cause gene-specific selective sweeps regardless of whether the loci subject to NFDS experience elevated recombination rates. One of these conditions is that high diversity is maintained at S loci within the population (i.e., *l*^*n*^ > > 1). Another condition is that NFDS is sufficiently stronger than directional selection on ecologically beneficial alleles (i.e., *s*_*i*_ > > *s*_*e*_) so that NFDS can oppose clonal expansion driven by directional selection (see ‘General framework of the model’; see also [42]). The third condition, which is implicitly assumed in the model, is that prokaryotic populations are sufficiently large (see Additional file 1 under ‘Effect of finite populations’), which apparently is a realistic assumption based on the available data [43].

### Can the conditions required for gene-specific selective sweep be satisfied in nature?

The available data indicate that the first and third conditions required for gene-specific selective sweeps, namely that there should be at least two loci subject to NFDS and that high diversity is maintained at these loci within the population, are likely to be satisfied in nature for virus-induced NFDS. In particular, metagenomic analyses indicate that genetic differences between strains of prokaryotes in the same habitat often are due, in part, to the presence or absence of genomic regions called metagenomic islands (MGIs). A typical prokaryote genome contains multiple (≤10) MGIs [27,44]. The MGIs usually consist of genes involved in the synthesis of extracellular components such as O-antigens and flagella, which can serve as targets for virus recognition [27]. Moreover, screening for virus-resistant mutants in a single bacterial culture has revealed many loci that carry resistance mutations to viruses [34]. Such resistance is effective against only a subset of tested viruses and commensurately increases the susceptibility of hosts to other viruses. In addition, these loci show considerable allelic diversity in the same habitat [34,45,46]. Taken together, these findings suggest that the number of loci involved in virus susceptibility in a typical prokaryote is substantially greater than one, and the number of possible susceptibility types is large.

The second condition for gene-specific selective sweeps is that recombination rates are sufficiently low. Whether or not this condition is satisfied in nature remains uncertain for two reasons. First, rates of spontaneous recombination in prokaryotes under natural conditions are unknown. Second, how low recombination rates must be is unclear because this depends on *α*, the factor by which recombination rates increase in the loci subject to NFDS, a value that is currently unknown and is likely to vary among prokaryotes.

The first problem can be partly addressed by inferring the rate of spontaneous recombination from the available data. For example, Overballe-Petersen et al. [47] conducted natural transformation assays to measure the spontaneous rate of recombination in *Acinetobacter baylyi* as a function of the concentration and size of extracellular DNA (eDNA). The eDNA concentrations in seawater particulates are on the order of 1 μg/ml [48] (note, however, that *A. baylyi* lives in soil). Assuming the most favorable conditions for recombination (namely, all eDNA is available for recombination at any given locus and is in the chromosomal length range), the recombination rate would be about 10^−3^ per cell per nucleotide per 90 min [47]. This value is likely to be an upper bound of recombination rates in this organism, and the realistic values should be at least a few orders of magnitude lower, i.e. about 10^−6^ to 10^−5^. At these rates, recombination alone would be too infrequent to cause gene-specific selective sweeps, given that a typical range of selection coefficients observed during experimental evolution is *s*_*e*_ ≥ 10^− 3^ [49]. However, whether or not NFDS can cause gene-specific selective sweeps under these conditions remains uncertain due to the uncertainty regarding *α*.

Finally, the condition that NFDS is sufficiently strong (i.e., *s*_*i*_ > > *s*_*e*_) is likely to be fulfilled in nature. Viral predation, likely the most prominent factor causing NFDS, is considered to be the major cause of prokaryote mortality in various natural environments. Virus-induced mortality (defined as cells killed by viruses per cell produced) is estimated to be >90% in seawater [50] and >80% in deep-ocean sediments [51]. A simple interpretation of these data is that viral predation can cause a selection coefficient of up to 0.9. Another factor that can cause NFDS is social interactions such as the production of public goods. In laboratory-generated cross-feeding bacterial consortia, NFDS is estimated to cause a selection coefficient of up to 0.14 [52] (calculated from the reported relative Malthusian fitness, following the method of [53]). These findings indicate that NFDS can cause selection coefficients (*s*_*i*_) of 0.1 or more. By contrast, the strength of directional selection due to ecologically beneficial alleles (*s*_*e*_) could be approximated by selection coefficients of beneficial mutations that arise during experimental evolution. In the majority of these experiments, the selection coefficient is estimated to be <0.1 [49]. Therefore, NFDS appears to be strong enough to oppose directional selection caused by ecologically beneficial alleles.

Overall, it does not seem unlikely that all the conditions required for NFDS to cause gene-specific sweeps are satisfied in nature although much ambiguity remains about the rates of spontaneous recombination. More experimental data are required to draw a stronger conclusion.

A possible experimental test for gene sweeps caused by NFDS might be sought in the fact that NFDS does not prevent genome-wide selective sweeps within each subpopulation. These restricted genome-wide sweeps would temporarily decrease the diversity of genotypes in the population without substantially decreasing per-locus neutral diversity. Although this signal would be eventually obliterated by recombination, it could be detectable soon after the sweep. If, alternatively, a gene-specific selective sweep were caused simply by exceedingly frequent recombination, a decrease in genotype diversity is not expected. Based on this difference, a test could be developed to distinguish between these two currently available hypotheses about the mechanism of gene-specific selective sweeps in prokaryotes.

### Comparison with previous studies

The model of gene-specific selective sweeps caused by NFDS is similar to the ‘adapt globally, act locally’ model of Majewski and Cohan [31], but differs from it in terms of the applicable scales of populations (see also [30] for related discussion). The previous model is concerned with selective sweeps across multiple, ecologically distinct populations of prokaryotes, a situation similar to trans-specific selective sweeps in sexual eukaryotes [54]. By contrast, here we considered selective sweeps in a population where the subpopulation structure is induced by NFDS, but the members of different subpopulations are frequently interchanged through recombination (at S loci), so that no permanent correlation exists between neutral polymorphisms and traits associated with subpopulations. The main point of the present study is that even in such an ecologically and genetically cohesive population, gene-specific selective sweeps can occur because of NFDS.

Mathematical models closely related to those analyzed here have been applied to eukaryotic populations by Peck [42], and Hodgson and Otto [41]. These previous studies investigate the advantage of recombination arising from interplay between NFDS and directional selection. Accordingly, they do not consider how these interactions affect neutral diversity, the question considered in the present study. This difference notwithstanding, the similarity between the models is striking and suggests wide applicability of the models incorporating interactions between NFDS and directional selection, which are likely to be common in complex ecosystems (see ‘Background’), for different aspects of evolution in prokaryotes and eukaryotes.

## Conclusions

The results of this modeling study indicate that NFDS is a realistic causative factor behind gene-specific selective sweeps in prokaryotes, provided recombination is sufficiently infrequent.

## Materials and methods

### Modeling host genotypes

To address the question posed in ‘Results’ (under ‘General framework of the model’), a population genetics model of evolving prokaryotes under NFDS was developed (our model is very similar to that described in Hodgson and Otto [41], except that it incorporates neutral loci, but does not assume modifier loci). Although NFDS can be caused by various types of ecological interactions, the model considered in this study assumes the kill-the-winner dynamics to incorporate NFDS for the sake of concreteness (an alternative model that incorporates NFDS without explicitly assuming host–parasite interactions is described in Additional file 1). Thus, the model considers populations of prokaryotic hosts and viruses. Prokaryotic genomes are assumed to encompass three types of loci (Figure 2b): (i) one E locus that assumes either the wild-type or beneficial allele (denoted by 0 and 1, respectively), (ii) *n* S loci that assume *l* alleles per locus (which determine susceptibility to viral infection as described later), and (iii) one N locus that assumes two neutral alleles (denoted by 0 or 1). For simplicity, the model explicitly incorporates only one N locus to consider the average relative decrease of per-locus neutral diversity caused by a selective sweep (per-locus diversity is more relevant than genotype diversity under the situation in which recombination is a more dominant source of genetic variation than mutations; also, per-locus diversity has been considered in previous work [17]). At S loci, there are a total of *l*^*n*^ allelic patterns (i.e., *l*^*n*^ susceptibility types). Thus, there are 4*l*^*n*^ host genotypes in total (each of which is represented by an integer denoted by *P*).

### Modeling fitness and virus genotypes

For simplicity, the interactions between the hosts and viruses are assumed to follow the matching-allele model [55,56]. Specifically, genomes of viruses consist of *n* loci each of which assumes *l* alleles (as in the S loci). If a viral genotype perfectly matches the allelic pattern of the host S loci, infection occurs. If there are mismatching loci, the probability of infection decreases exponentially with the number of such loci (denoted by ${d}_{S_P,V}$). Under these assumptions, the fitness of a host genotype *P* was defined as

$$ {f}_P=\left(1+{s}_e{E}_P\right){\displaystyle \sum_{V=0}^{l^n-1}\left[1-{s}_i exp\left(-{d}_{S_P,V}\right)\right]{p}_V}. $$

The expression in the first bracket reflects the effect of the E locus, which increases fitness by *s*_*e*_ if the genome carries the beneficial allele (see Table 1 for notation). The expression in the second bracket under the sum reflects the effect of the S loci, which decreases fitness by at most *s*_*i*_ depending on the frequency of viruses *p*_*V*_ and the probability of infection. In a similar fashion, the fitness of a virus genotype *V* is defined as

$$ {f}_V={\displaystyle \sum_{P=0}^{4{l}^n-1} exp\left(-{d}_{S_P,V}\right){p}_P}. $$

The value of *s*_*e*_ was set to 0.01 because selection coefficients of beneficial mutations that arise during experimental evolution are estimated to be between 10^−3^ and 10^−1^, which might approximate selection pressure due to ecologically beneficial alleles. The value of *s*_*i*_ was set to 0.1 given the high virus-induced mortality of prokaryotes in marine environments [50,51], which suggests that *s*_*i*_ is much higher than *s*_*e*_.

### Modeling recombination

The rate of recombination is likely to depend on various factors including those that affect an entire genome such as mismatch-repair activity and those that affect specific loci such as the presence of site-specific recombinases. In the model, these effects are assumed to be absorbed into two parameters, *r* and *α. r* is the genome-wide, basal rate of recombination per locus per generation. *α* is a factor by which the basal rate is modified by locus-specific factors. At the E and N loci, recombination was assumed to occur at the rate *r*. At the S loci, this rate was increased by a factor *α* to take account of the assumed high mobility at these loci (*α* > > 1 unless otherwise stated).

Recombination replaces the allele at the affected locus with an incoming allele (like gene conversion), which is determined by the frequencies of alleles in the source of the DNA for recombination. At the E and N loci, recombining DNA is assumed to originate exclusively from within the given population to consider the condition most unfavorable for a gene-specific selective sweep. Accordingly, the frequencies of incoming alleles were set identical to the frequencies of the alleles in the given population. At the S loci, incoming DNA was assumed to originate from a large, exogenous source. Accordingly, the frequencies of incoming alleles were set to *l*^−1^. This assumption increases the chance of introducing rare alleles at the S loci and thus amounts to high mobility at these loci.

Finally, mutations were ignored because we analyze the situation in which recombination is the dominant source of genetic variation (the model incorporating mutations is described in Additional file 1 under ‘Effect of finite populations’). In viruses, alleles are replaced by one of the *l* alleles with an equal frequency at a rate *r*_*V*_ per locus per generation (whether this is due to recombination or mutation is irrelevant to this study and thus unspecified).

### Modeling population dynamics

The population size was assumed to be infinitely large (the model assuming finite populations is described in Additional file 1 under ‘Effect of finite populations’). The recombination-selection dynamics was defined by the difference equations given in Additional file 1.

### Simulations

Under the model defined above, the following simulations were performed to evaluate the effect of NFDS on the mode of fixation of beneficial alleles. First, the model was initialized by randomizing the frequencies of the host and virus genotypes. During this initialization, the frequency of the E alleles was set at zero, and the frequencies of the two N alleles were set equal to each other. The first condition implies that a selective sweep has not yet started to occur in the host population. The second implies that the clonality at the N locus (denoted by *J*) was a minimum before the selective sweep. The clonality was defined as

$$ J={p}_N^2+{\left(1-{p}_N\right)}^2, $$

where *p*_*N*_ is the frequency of allele 1 at the N locus [57]. Then, the simulation was run for a number of steps (4 × 10^4^ generations) to eliminate transient effects. Subsequently, the beneficial allele was introduced as follows. One host genotype was arbitrarily chosen, and a small fraction of it (viz., 10^− 9^) was converted into an adaptive genotype by replacing the wild-type allele at the E locus with the beneficial allele. Then, the simulation was continued until the frequency of the beneficial allele was increased to a high value (0.99), at which point the allele was considered fixed. After the fixation, the clonality *J* was measured (denoted by *J*_*a*_; averaged over 2,000 generations to remove the effect of oscillations). If a genome-wide selective sweep occurred, *J*_*a*_ would increase to unity; conversely, if a gene-specific selective sweep occurred, *J*_*a*_ would remain at the original value before the selective sweep (denoted by *J*_*b*_). Thus, the relative increase of clonality caused by fixation of the beneficial allele was defined as

1 $$ {J}_{\mathrm{rel}}=\frac{J_a-{J}_b}{1-{J}_b}. $$

*J*_rel_ assumes values between 0 and 1, ranging from the case of a gene-specific selective sweep to the case of a genome-wide selective sweep, respectively.

## Additional file

Additional file 1: **Supplementary materials and methods, and results.**

## Abbreviations

eDNA: extracellular DNA
E locus: ecological locus
MGI: metagenomic island
NFDS: negative frequency-dependent selection
N locus: neutral locus
S locus: susceptibility locus
