# Supplementary material for: Gene-specific selective sweeps in bacteria and archaea caused by negative frequency-dependent selection
Source: BMC Biol. 2015 Apr 16;13:20. doi: 10.1186/s12915-015-0131-7 (PMC4410459; doi:10.1186/s12915-015-0131-7)
Supplement: Supplementary file 1 — Supplementary materials and methods, and results. [file 12915_2015_131_MOESM1_ESM.pdf]

Supplementary materials and methods

Equations defining the model

The population dynamics of prokaryotic hosts is defined by the following selection $\mathbf{S}$ and recombination matrix $\mathbf{R}$:

$$[\mathbf{S}]_{P,P} = \frac{f_P}{\langle f_P \rangle_t},$$

$$[\mathbf{R}]_{P,P'} = \rho_{P,P'},$$

$$\vec{p}(t+1) = \mathbf{R}\mathbf{S}\vec{p}(t).$$

$\langle f_P \rangle_t$ is the average fitness of prokaryotic hosts at time t. $p_P(t)$ is the frequency of prokaryote genotype P at time t. $\vec{p}(t)$ is the vector whose P^{th} entry is $p_P(t)$. $\mathbf{S}$ is a diagonal matrix (the entry at the ith row and the jth column of a matrix $\mathbf{X}$ is denoted by $[\mathbf{X}]_{i,j}$). $\rho_{P,P'}$ is the rate of recombination that transforms prokaryote genotype P' into P, and it is defined as

$$\begin{aligned}
\rho_{P,P'} &= (\alpha r / a)^{d_{S_P,S_{P'}}} (1-\alpha r / a)^{n-d_{S_P,S_{P'}}} \\
&\times \{\delta_{E_P,E_{P'}}[1-\delta_{E_P,0} r p_E(t) - \delta_{E_P,1} r(1-p_E(t))] + (1-\delta_{E_P,E_{P'}}) r[\delta_{E_P,1} p_E(t) + \delta_{E_P,0}(1-p_E(t))]\} \\
&\times \{\delta_{N_P,N_{P'}}[1-\delta_{N_P,0} r p_N(t) - \delta_{N_P,1} r(1-p_N(t))] + (1-\delta_{N_P,N_{P'}}) r[\delta_{N_P,1} p_N(t) + \delta_{N_P,0}(1-p_N(t))]\}
\end{aligned}$$

where $\delta_{i,j}$ is Kronecker's delta, $d_{S_P,S_{P'}}$ is the number of S loci that differ between P and P', and $p_E(t)$ and $p_N(t)$ are the frequency of allele 1 at the E locus and that at the N locus at time t, respectively.

The population dynamics of viruses is similarly defined: $\mathbf{S}$ is basically identical to the above with P replaced by V, and the entries of $\mathbf{R}$ is defined as

$$\rho_{V,V'} = (r_v / a)^{d_{V,V'}} (1-r_v / a)^{n-d_{V,V'}}.$$

Supplementary results

Maximum recombination rate below which NFDS can cause gene-specific selective sweeps

As described under Results, gene-specific selective sweeps require sufficiently low basal recombination rates (Figure 3c). This result was interpreted based on the inequality $(\alpha r)^2 s_e^{-1} << r$. Below, we derive a similar inequality based on simplified mathematical models.

As described in Results section, the beneficial allele at the E locus spreads through the two scenarios: the pathway involving recombination at the E locus and the pathway involving recombination at the S loci. We can evaluate which pathway allows faster spread and, therefore, is more dominant than the other by considering simplified mathematical models that describe the spread of the beneficial allele either via the one or the other pathway.

The spread of the allele via the pathway involving recombination at the E locus can be modeled by the equation

$$\frac{dx_i}{dt} = s_e x_i + r x_0 , \qquad \text{(S1)}$$

where x_i is the frequency of individuals carrying the beneficial allele in one subpopulation, assuming that x_i is small. The index i denotes different subpopulations. x_0 is the frequency of the individuals that already have the beneficial allele (x_0 is assumed to be constant). rx_0 is the rate of production of x_i via recombination at the E locus. This same term appears in the equation for any value of i because production of x_i via this pathway requires only one recombination event.

By contrast, the spread of the allele via the pathway involving recombination at the S loci can be modeled by the equation

$$\frac{dy_i}{dt} = s_e y_i + \alpha r y_{i-1} , \qquad \text{(S2)}$$

where y_i is the frequency of individuals carrying the beneficial allele in one subpopulation, assuming that y_i is small. y_0 is assumed to be constant (as is the case for x_0). $\alpha r y_{i-1}$ is the rate of production of y_i via the transformation of y_{i-1} through

recombination at the S loci. We assumed that y_{i-h} can be transformed into y_i by h recombination events at the S loci. Moreover, we ignored the terms of the order $o(r)$ for $r \to 0$ as well as the decrease of y_i due to transformation into y_{i+1}. Note the difference between Eqs. (S1) and (S2): x_i can be reached from x_0 by one recombination step, whereas y_i requires at least i recombination steps from y_0.

Using the above equations, the speed at which the beneficial gene spreads through the ith subpopulation can be estimated as the times required for x_i and y_i to reach a high value starting at 0. These estimates need not be precise—the only information needed is how these times depend on the two parameters, r and n. Eq. (S1) can be integrated:

$$x_i = \frac{r}{s_e} x_0 \, \mathrm{e}^{s_e t}(1 - \mathrm{e}^{-s_e t}), \qquad \text{(S3)}$$

where we set $x_i = 0$ at $t = 0$. Considering only the highest term in x_i for $t \to \infty$, we can approximate the time (denoted by τ_E) at which x_i reaches an arbitrary value $\bar{x}$ by

$$s_e \tau_E \approx \ln \frac{s_e}{r} + \ln \frac{\bar{x}}{x_0}. \qquad \text{(S4)}$$

Thus, τ_E depends logarithmically on r.

For the pathway involving recombination at the S loci, Eq. (S2) can be integrated for $i = 1$ to obtain

$$y_1 = \frac{\alpha r}{s_e} y_0 \, \mathrm{e}^{s_e t}(1 - \mathrm{e}^{-s_e t}),$$

which is, of course, the same as Eq. (S3) with r replaced by αr. Because $\alpha >> 1$, the time (denoted by τ_1) at which y_1 reaches an arbitrary value $\bar{y}$ is always shorter than τ_E (assuming that $y_0 = x_0$ and $\bar{y} = \bar{x}$). For $i = 2$, substituting the above equation in Eq. (S2) yields

$$\frac{dy_2}{dt} = s_e y_2 + \alpha r \frac{\alpha r}{s_e} x_0 \, \mathrm{e}^{s_e t}(1 - \mathrm{e}^{-s_e t}).$$

This can be integrated:

$$y_2 = \frac{\alpha^2 r^2}{s_e^2} x_0 \, \mathrm{e}^{s_e t}(s_e t - 1 + \mathrm{e}^{-s_e t}).$$

Considering only the highest order term, we can approximate the time at which y_2 reaches a value $\bar{y}$ (denoted by τ_2) by

$$s_e \tau_2 \approx \ln \frac{s_e}{(\alpha r)^2 \tau_2} + \ln \frac{\bar{y}}{y_0}. \qquad \text{(S5)}$$

Taking derivative with respect to r, we obtain

$$\frac{\partial \tau_2}{\partial r} \approx -\frac{2\tau_2}{(s_e \tau_2 + 1) r} \sim -\frac{2}{s_e r} \quad (\text{as } r \to 0),$$

where we used the fact that $\tau_2 \to \infty$ as $r \to 0$. The last equation indicates that τ_2 depends logarithmically on r as $r \to 0$. From Eqs. (S4) and (S5), we obtain (assuming that $y_0 = x_0$ and $\bar{y} = \bar{x}$)

$$s_e(\tau_2 - \tau_E) \approx \ln \frac{r}{(\alpha r)^2 \tau_2} \to \infty \quad (\text{as } r \to 0), \qquad \text{(S6)}$$

where we used the fact that τ_2 depends logarithmically on r as $r \to 0$. According to Eq. (S6), if r is so small that $(\alpha r)^2 \tau_2 << r$, the pathway involving recombination at the E locus becomes dominant over the pathway involving recombination at the S loci. τ_2 depends only logarithmically on r, so that it does not much affect the order-of-magnitude comparison between $(\alpha r)^2 \tau_2$ and r. Numerical calculations show that $\tau_2 >> s_e^{-1}$ (data not shown).

Effect of finite population sizes

In the above argument, the logarithmic dependency of τ_E and τ_2 on r is crucial. For example, if the dependency of τ_E and τ_2 were inversely linear such that $\tau_E = r^{-1}$ and $\tau_2 = 2(\alpha r)^{-1}$, the difference is $\tau_2 - \tau_E = (2\alpha^{-1} - 1) r^{-1}$, which is always negative for any values of r since $\alpha >> 1$. This fact implies yet another condition that must be satisfied for NFDS to cause gene-specific selective sweeps. Namely, the census size of a prokaryote population must be sufficiently large. To see why this condition is required, let us consider a finite population of size M. If M is sufficiently large, the dynamics of the population is approximated by the infinite population model. If, however, M is so small that the recombination rate per population (rM) is smaller than unity, such approximation is no longer valid. In this case, the spread of the beneficial allele is limited by the occurrence of rare recombination events. The expected waiting time for

recombination and accordingly for the spread of the beneficial allele is inversely proportional to r. In this case, in contrast to the infinite population model, a gene-specific selective sweep at low r is not expected.

To test the above argument, a finite population model was constructed. The model was formulated as a Fisher-Wright process with asexual, haploid genomes. The fitness was defined as in the infinite population model. Per-generation probabilities of recombination were defined in the same manner as per-generation rates of recombination in the infinite population model. Using this model, the relative increase of clonality J_{rel} was measured as a function of population size M. The results show that as M increases, J_{rel} gradually decreases toward the values predicted by the infinite population model (Figure S1). This result indicates that gene-specific selective sweeps require that M be sufficiently large, in agreement with the above argument.

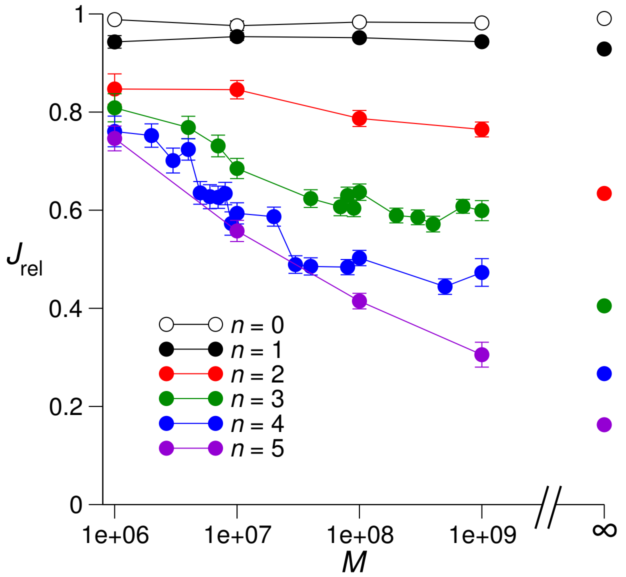

Figure S1 Relative clonality J_{rel} as a function of host population size M. In the finite population model, mutations are necessary to maintain diversity at the N locus in the face of genetic drift; thus, a mutation rate of 10^{-6} per generation was assumed at the N locus. To speed up simulations, population sizes of viruses are assumed to be the same as those of the hosts. The data for $M = \infty$ were obtained with the infinite population model. Error bars indicate standard error of mean (applicable only to the finite

population model). The sample size was 100 per data point. The parameters were as follows: $l = 2$, $\alpha = 10$, $r = 10^{-6}$, $s_e = 0.01$, $s_i = 0.1$, $r_v = 10^{-4}$.

Alternative model

The model described in the main text assumes the kill-the-winner dynamics caused by viruses to incorporate NFDS. We focused on this model because the kill-the-winner dynamics seems to be the predominant mechanism that causes NFDS and high diversity in natural prokaryotic populations. However, the kill-the-winner dynamics per se is not necessary for NFDS because NFDS can be caused by other types of ecological or social interactions (see Introduction). Moreover, multiple mechanisms of NFDS might operate concurrently within a natural prokaryotic population.

To examine whether NFDS can cause gene-specific selective sweeps regardless of the specific mechanisms that cause NFDS, we considered an alternative model that abstracts away from the kill-the-winner dynamics and also takes account of multiple, concurrently-operating mechanisms for NFDS. This model assumes that prokaryote genomes have multiple S loci subject to NFDS that do not interact with each other (i.e., NFDS imposed at different loci is caused by independent mechanisms). Specifically, the fitness of prokaryotes was defined in a frequency-dependent manner as follows:

$$f_P = \left(1 + s_e E_P\right) \prod_{k=1}^{n} (1 - s_i p_{P_{S_k}}),$$

where $p_{P_{S_k}}$ is the frequency of the allele at the k th S locus of the host genome P in the prokaryote population. The population dynamics of other biological entities such as viruses is not explicitly incorporated into the model. This model is very similar to the model considered in Peck [1], except that the latter does not consider neutral loci.

The results obtained with the above alternative model showed that NFDS can cause gene-specific sweeps even if the recombination rate at the loci subject to NFDS is higher than at the other loci (E and N), provided the conditions described in the main text are satisfied (Figure S2). These results suggest that NFDS can cause gene-specific selective sweeps regardless of the specific mechanisms that lead to NFDS.

There is also a difference between the results obtained with the alternative model and those obtained with the model described in the main text. As shown in Figure

S2b, the value of J_{rel} for $n=5$ does not become as small as that for $n=2$ as $r \to 0$ in contrast to the case for the original model (Figure 3c), where J_{rel} for $n=5$ become as small as that for $n=2$ as $r \to 0$. The reason for this discrepancy is as follows. The restriction of genome-wide selective sweeps (and, thereby, the promotion of gene-specific selective sweeps) depends on the maximum fraction a particular susceptibility type can attain within a prokaryote population as described in Result section. In the original model, this fraction is l^{-n}. In the alternative model, it is l^{-1} because each S locus is subject to NFDS independently of each other. Because l was set larger for $n=2$ than for $n=5$ in Figure S2b (so as to be consistent with Figure 3c), the minimum possible value of J_{rel} is smaller for $n=2$ than for $n=5$.

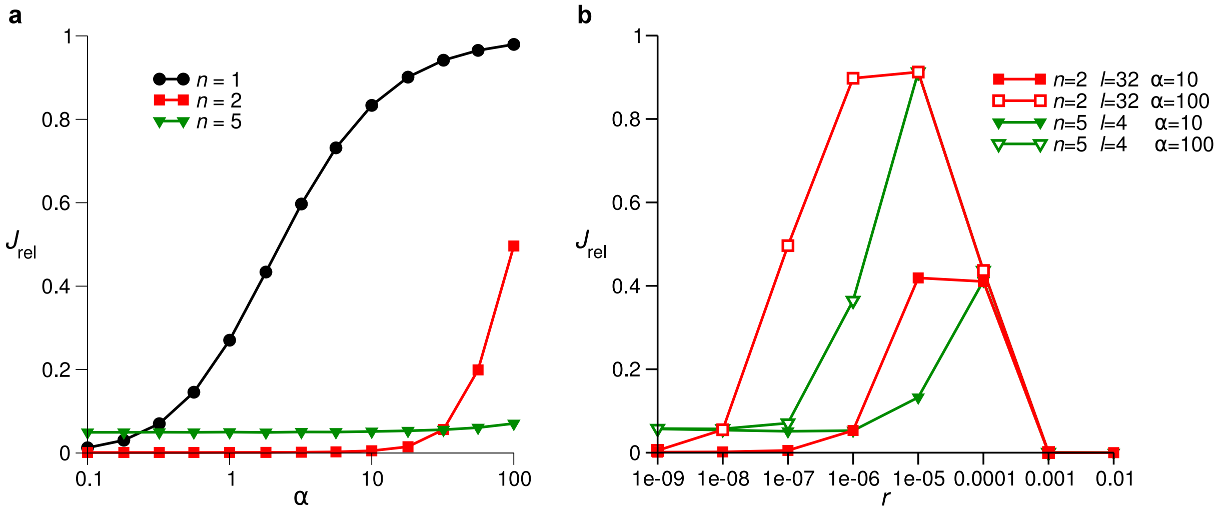

Figure S2 Relative clonality measured with the alternative model. **(a)** Relative clonality J_{rel} as a function of α (see Table 1 for notation). l^n was fixed at 1024, while n was varied as indicated in the graph. The other parameters were as follows: $r=10^{-7}$, $s_e=0.01$, $s_i=0.05$, and $r_v=10^{-4}$. **(b)** Relative clonality J_{rel} as a function of r. l^n was fixed at 1024, while n, l, and α were varied as indicated in the graph. The other parameters were as follows: $s_e=0.01$, $s_i=0.05$, and $r_v=10^{-4}$.

References

1. Peck JR: **Frequency-Dependent Selection, Beneficial Mutations, and the Evolution of Sex**. *Proc R Soc Lond B Biol Sci* 1993, **254**:87–92.
